# Comparison between Online and Offline Price of Tobacco Products Using Novel Datasets

**DOI:** 10.3390/ijerph15102282

**Published:** 2018-10-17

**Authors:** Magdalena Opazo Breton, John Britton, Yue Huang, Ilze Bogdanovica

**Affiliations:** Division of Epidemiology and Public Health, Univeristy of Nottingham/UK Centre for Tobacco and Alcohol Studies, Clinical Sciences Building, City Hospital, Hucknall Road, Nottingham NG5 1PB, UK; Magdalena.Opazo@nottingham.ac.uk (M.O.B.); j.britton@outlook.com (J.B.); Yue.Huang@nottingham.ac.uk (Y.H.)

**Keywords:** cigarettes, prices, tobacco

## Abstract

Price of tobacco products has traditionally been relevant both for the industry, to respond to policy changes, and for governments, as an effective tobacco control measure. However, monitoring prices across a wide range of brands and brand variants requires access to expensive commercial sales databases. This study aims to investigate the comparability of average tobacco prices from two commercial sources and an in-house monitoring database which provides daily data in real time at minimal cost. We used descriptive and regression analysis to compare the monthly average numbers of brands, brand variants, products and prices of cigarettes and hand-rolling tobacco using commercial data from *Nielsen Scantrack* and *Kantar Worldpanel*, and an online price database (OPD) created in Nottingham, for the period from May 2013 to February 2017. There were marked differences in the number of products tracked in the three data sources. Nielsen was the most comprehensive and Kantar Worldpanel the least. Though average prices were very similar between the three datasets, Nottingham OPD prices were the highest and Kantar Worldpanel the lowest. However, regression analysis demonstrated that after adjustment for differences in product range, price differences between the datasets were very small. After allowing for differences in product range these data sources offer representative prices for application in price research. Online price tracking offers an inexpensive and near real-time alternative to the commercial datasets.

## 1. Introduction

Over recent years comprehensive tobacco control policies [1] have reduced smoking prevalence in the United Kingdom to a new low of 15.1% in 2017 [2], but further measures are needed to reduce the current total of 7.4 million adult smokers at risk of premature death and disability caused by smoking [2]. Using taxation to increase the price of tobacco is one of the most effective means of achieving this, as higher prices reduce smoking uptake, increase smoking cessation, and also reduce social inequalities in smoking [3,4]. However the tobacco industry is adept at managing prices in response to policy changes [5], so it is important to be able to access reliable data across a wide spectrum of tobacco products easily, cheaply, and ideally in real time. Although a range of data sources and metrics have been used to this end, including the Most Popular Price Category [6] and Weighted Average Price [7], prices published in a retail newsagent magazine [7], and national and international surveys of self-reported purchase prices [8,9], comprehensive data on individual tobacco products are available only from commercial sources. The most widely used of these is Nielsen Scantrack [7], which measures sales at bricks-and-mortar (but not online) retailer checkouts; while an alternative that has been less widely used for tobacco research is Kantar Worldpanel [10,11], which collects data on products purchased, including online, by a panel of households. 

Both of these sources provide data in extensive detail but at appreciable financial cost to the user. Neither provides data in real time. We set up a database to record tobacco prices, based on extracting price data for tobacco products listed on a supermarket price comparison website. The aim of this study was to explore the comparability of tobacco price data obtained from three sources: two commercial organisations providing data retrospectively at cost, and Nottingham OPD data downloaded without charge from online sources. 

## 2. Materials and Methods

### 2.1. Data

Price data were obtained from Nielsen Scantrack, Kantar Worldpanel and our own online price database (Nottingham OPD). 

Nielsen Scantrack estimates average monthly prices using value of sales and units sold from over 75,000 bricks-and-mortar retail stores in United Kingdom, including megastores, superstores, high street stores and convenience stores [7,12]. From March 2013 the dataset subcategorized product prices as standard or promotional, the latter being identified by a price drop of 5% or more relative to the second highest price registered for the product in the six previous weeks. 

Kantar Worldpanel is a shoppers panel that includes around 30,000 households geographically and demographically representative to the population of Great Britain [13]. On average every month a panel of 1800 households with at least one smoker scanned their purchased tobacco products, both from bricks-and-mortar retailers and online. For the purpose of this study Kantar Worldpanel data included only products that were purchased and taken back into the home. Products that were bought and then used or consumed on-the-go were not included. For this reason there would be some expected differences between Kantar Worldpanel data and retailer sales data.

Prices were also categorized as promotional or standard (based on the product receipt), but represent actual price paid (receipt price) rather than an estimate based on sales volumes. 

The Nottingham OPD collected daily prices from an online price comparison website (www.mysupermarket.co.uk) which includes products sold at all major bricks-and-mortar and online supermarkets in the UK including Tesco, Asda, Ocado, Waitrose, Sainsbury’s and Morrisons. The price data were typically updated daily [14], and we recorded the data every day. Webpages were downloaded automatically using the Python Selenium library [15], and the Python Beautiful Soup library [16] to parse the html elements to extract product name and price information into a local database.

### 2.2. Measures

To compare prices across the different data sources we synchronized product labels (for example, the same product was listed as Windsor Blue in one dataset and Windsor Blue JPS in another) to describe the same products across all datasets. As previously described [17], cigarette products were defined by a unique combination of brand (for example Pall Mall, Marlboro, Dunhill), brand variant or descriptor (for example, superkings, red, yellow, capsule), pack size (number of cigarettes per pack) and multipack size (number of packs). Hence, one example of a cigarette product would be a single pack of 20 Dunhill International cigarettes; another a multipack of 5 packs of 18 Pall Mall Superkings Blue Capsule Cigarettes. Hand-rolling tobacco products were defined by a unique combination of brand name (for example Cutters Choice, Drum), brand variant or descriptor (gold, handy pack, blonde), pack volume (weight in grams) and multipack size (number of packs per multipack).

The data sources were made comparable geographically by removing Northern Ireland from Nielsen data and in terms of trade channel (retail or online) by dividing Kantar Worldpanel data into online and conventional (bricks-and-mortar) retail. Hence, we were able to compare retail prices from Kantar Worldpanel and Nielsen, and online prices from Kantar Worldpanel and the Nottingham OPD. 

Our outcome variables were monthly average numbers of brands, brand variants, products and prices separately for manufactured cigarettes and hand-rolling tobacco. Since products were defined by a combination of brand, brand variant, pack size and multi pack size, to compare the average number of products across dataset we categorized products by multipack size in two categories (single pack or multi pack) and by pack size in four categories for both cigarettes (10 cigarettes, 11–19 cigarettes, 20 cigarettes and more than 20 cigarettes) and hand-rolling tobacco (less than 12.5 grams, 12.5 grams, 13–29 grams and 30 gram or larger packs). For equivalence with the Nielsen and Kantar sources we calculated monthly average prices for the Nottingham OPD as arithmetic means of the prices charged by all retailers offering each specific product. Prices were then expressed as price per cigarette for cigarettes and price per gram for hand-rolling tobacco. We cleaned the datasets by deleting those products that had less than four observations throughout the period studied, and we omitted price outliers by deleting those prices that increased by more than 200% for the same product between consecutive observations. We analysed data for the period for which data were available from all three data sources, from May 2013 to February 2017. 

### 2.3. Statistical Analysis

The numbers of brands, brand variants and products in each dataset were calculated for each month and summarised as an overall average. Monthly average prices from each dataset were plotted against time and compared as gross figures for all products present in each dataset, and for the subset of products present in all datasets. 

Since price per cigarette and price per gram exhibited an approximately normal distribution in a histogram, a linear regression model was used to compare prices from the sources that have not yet been used in tobacco price research (Kantar Worldpanel and Nottingham OPD), to the most frequently used source of prices in tobacco research, Nielsen Scantrack (Nielsen was the reference category in our regression). Adjusted mean difference between Kantar and Nottingham OPD compared to Nielsen were obtained using two specifications of the model. The first model was adjusted by pack size, year and month to obtain adjusted means accounting for the fact that price is determined by pack size and time trends. The second model was adjusted for year, month and product to account for the fact that we had panel data on cigarettes and hand-rolling tobacco products. All analysis was done using Stata 15 and the statistical significance level was set at 0.05.

## 3. Results

### 3.1. Numbers of Products with Data

There were marked differences between the Nielsen, Kantar retail, Kantar online and Nottingham OPD datasets in terms of monthly average number of brands, brand variants and products, both for cigarettes and hand-rolling tobacco products (Table 1). Across the full range of brands, brand variants and products in all single and multipack size categories Nielsen retail data typically provided the highest average monthly numbers, Nottingham OPD the second highest, and Kantar retail the lowest. For example, the average number of cigarette products in single packs in the Nielsen dataset was 185.0 (95% CI 182.0 to 187.9), in Nottingham OPD 134.1 (95% CI 129.8 to 138.4), in Kantar retail 99.6 (95% CI 97.9 to 101.2), and in Kantar online 47.4 (95% CI 42.9 to 51.9). More marked differences in numbers were evident for brand variant and pack size categories (Table 1). The distribution of various product sizes for single packs of cigarettes also differed between data sources, with packs of 20 cigarettes being the most frequent in the Nielsen data, and products in packs of 10-19 the most frequent in the other data sources. For hand-rolling tobacco however, pack size distribution was broadly similar in all datasets (Table 1). 

### 3.2. Prices

**Figure 1:**
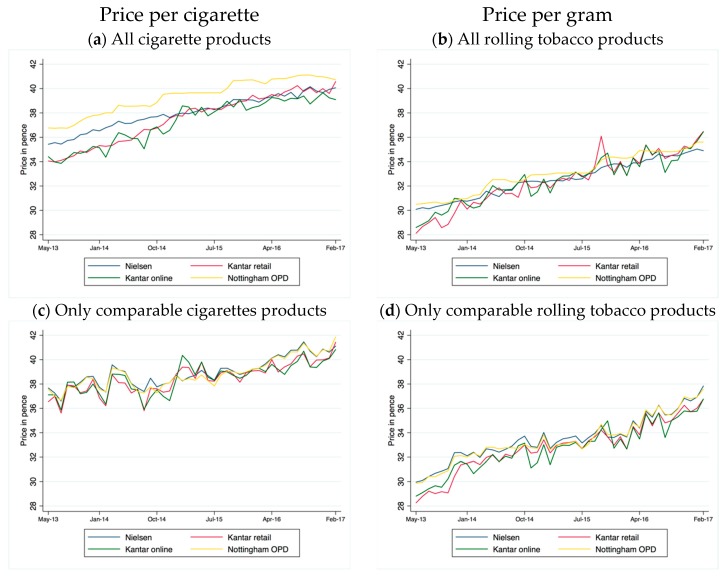
Distribution of monthly average price: per cigarette for cigarettes and per gram for rolling tobacco by data source (all products and only comparable products).

Average prices per cigarette over the whole study period differed significantly between datasets, ranging from 34.9 (95% CI 34.1 to 35.8) and 35.2 (95% CI 34.3 to 36.1) pence in Kantar online and retail data to 38.0 (95% CI 37.6 to 38.5) in Nielsen and 39.8 (95% CI 39.4 to 40.2) in Nottingham OPD data (Table 1). A similar trend applied for hand-rolling tobacco prices, which ranged from 30.5 (95% CI 29.7 to 31.2) in Kantar retail to 33.6 (95% CI 33.1 to 34.1) in Nottingham OPD data. Analysis of trends in average price over time indicate however that for cigarettes in particular, Nottingham OPD prices were consistently higher while those from the other sources tended, from 2015 onwards, to converge (Figure 1a). The gap between Nielsen and Nottingham OPD was fairly constant throughout the study period at around 1.3 pence (range 0.7 to 2.0 pence). Time trends in price were relatively similar between data sources for hand-rolling tobacco (Figure 1b), though prices per gram tended to be more variable for Kantar retail and Kantar online (Table 1), probably because the number of products was relatively small.

However, trends in prices of comparable products (that is, products listed in all datasets) were very similar in all datasets (Figure 1c,d) and average prices slightly higher, indicating that differences in overall average prices are likely to be attributable to differences in the range of products for which data are available in each dataset, with a relatively high proportion of low-price (particularly packs of 10–19 cigarettes) in the Kantar data.

### 3.3. Comparing Data Sources Using Regression Analysis

Our regression results for price per cigarette demonstrate that in relation to Nielsen data, Kantar (retail and online) and Nottingham OPD prices showed modest, though in several cases highly significant, differences in price per cigarette or gram of hand-rolling tobacco, with Kantar data tending to be lower and Nottingham OPD data higher than Nielsen figures (Table 2). In the fully adjusted model (Model 2) these differences ranged from −0.008 pence to no difference for cigarettes and from −0.247 to 0.096 for rolling tobacco (Table 2).

## 4. Discussion

This study compares, for the first time, cigarette and hand-rolling tobacco prices from two independent commercial datasets and a bespoke price tracking database which monitors online supermarket prices. Our findings demonstrate marked differences in the number of products tracked in these sources, with Nielsen including the most and Kantar the least, and that average prices for the same products were very similar in all data sources. Therefore, whilst overall average prices across the full range of products in each dataset tended to be higher for the Nottingham OPD and lowest for Kantar data, these differences arose from differences in the products tracked. In particular, Kantar captured a relatively high proportion of packs of 10–19 cigarettes, which are predominantly budget brands, while the other data sources featured higher proportions of 20 packs and hence more premium brands. Generally, the number of cigarette products was higher than that for hand-rolling tobacco. After adjustment for pack size, time and product fixed effects, differences in prices for the same products were very modest, as were differences in retail and online prices, and similarly to findings from previous research [17] we did not observe seasonal trends in prices. It is possible that the differences observed in average prices are to some extent due to regional variations though online dataset does not allow to explore prices at regional level.

These findings indicate that for any given product, retail and online prices tend to be similar and all of these datasets offer representative prices for application in price research. However the differences between them are also important, with Kantar representing a higher proportion of low-price brands that were, in the two years leading up to the implementation of standardized packaging in the UK, increasingly packaged in packs of less than 20 cigarettes [17]. It is possible that this difference arises from the fact that Kantar data are collected from customers scanning their purchased products, whereas Nielsen data include all sales in monitored retail outlets and Nottingham OPD only published prices, not sales; in which case the implication is that the Kantar panel includes a higher proportion of price-sensitive smokers who minimize the price they pay for tobacco [18], which if true may arise from socioeconomic bias in willingness to engage in the regular scanning of purchases demanded by this system. Previous research using Kantar data has demonstrated that the panel contains a higher proportion of middle aged, multiple adult households than national surveys [19], though we are unable to determine whether this characteristic alone explains our findings. A further potential explanation for modestly lower average prices in the Kantar data is that not all household purchases are necessarily scanned, raising the possibility that unscanned products tend to be purchased at a higher price. It is also important to explore whether comparability of datasets changes with the full implementation of standardized packaging legislation once all relevant data are available. 

## 5. Conclusions

After allowing for differences in purchase patterns however, our findings demonstrate that these three datasets offer very similar estimates of prices, particularly when restricted product range is used; and therefore that the choice of dataset should be determined by their other characteristics. The advantages of online tracking are that it is relatively inexpensive and can provide real-time price data, but with the disadvantage that no measure of sales is available. Nielsen is the most extensive source in terms of number of products and can also provide sales volume data, making it perhaps the most suitable source to monitor market developments. In contrast, Kantar offer a unique opportunity to explore what individual households are actually paying for tobacco products, and also provide consumption data. Our study thus indicates that for simple monitoring of brand diversity and price, online tracking is adequate and that the Nottingham OPD (data available on request) provides price estimates that can be used as a cost-free alternative when information on tobacco prices is required. For more detailed measures of consumption and household purchase patterns, both Nielsen and Kantar offer different but extensive data. 

## Figures and Tables

**Table 1:** Product diversity within and between the four data sources: brand, brand variants, pack size and multi-pack size for cigarette and rolling tobacco products.

		Nielsen (Retail)	Kantar Worldpanel (Retail)	Kantar Worldpanel (Online)	Nottingham OPD (Online)
Average monthly number of brands (95% CI)	Cigarettes	41.8 (41.2–42.5)	27.8 (27.1–28.5)	20.3 (19.7–20.8)	32.8 (32.5–33.1)
	Rolling tobacco	19.5 (19.2–19.8)	10.5 (10.1–10.9)	10.4 (10.0–10.8)	17.9 (17.2–18.5)
Monthly average number of brand variants (95% CI)	Cigarettes	185.4 (182.4–188.4)	102.5 (100.1–104.1)	58.3 (54.4–62.2)	134.8 (130.4–139.3)
	Rolling tobacco	34.6 (33.3–35.9)	10.9 (10.5–11.3)	10.8 (10.4–11.2)	23.9 (22.6–25.1)
Average monthly number of products by multipack size (95% CI)	Cigarettes				
	Single pack	185.0 (182.0–187.9)	99.6 (97.9–101.2)	47.4 (42.9–51.9)	134.1 (129.8–138.4)
	Multi pack	67.6 (65.9–69.3)	41.9 (40.5–43.2)	26.4 (25.3–27.4)	53.2 (52.0–54.4)
	Rolling tobacco				
	Single pack	90.4 (86.2–94.7)	21.6 (20.8–22.5)	21.4 (20.5–22.2)	52.1 (49.2–54.9)
	Multi pack	0.4 (0.3–0.6)	0	0	0
Average monthly number of products by pack size, only single pack (95% CI)	Cigarettes				
	10 cigs.	65.9 (64.4–67.4)	36.8 (35.7–38.0)	5.8 (4.4–7.2)	45 (43.8–46.2)
	11–19 cigs.	109.2 (94.8–123.5)	61.8 (55.3–68.4)	28.5 (24.0–33.0)	86.8 (78.5–95.2)
	20 cigs.	153.1 (149.6–156.5)	51.8 (47.4–56.2)	19.2 (16.7–21.7)	61.1 (57.4–64.9)
	>20 cigs.	4.3 (4.1–4.5)	4.7 (3.8–5.6)	0.5 (0.3–0.7)	2.2 (1.9–2.6)
	Rolling tobacco				
	<12.5 gr.	13.4 (11.7–15.2)	1.6 (1.2–2.0)	1.5 (1.1–1.8)	6.3 (5.5–7.2)
	12.5 gr.	27.3 (26.8–28.7)	3.4 (3.1–3.7)	3.3 (3.0–3.7)	12.8 (12.5–13.1)
	13–29 gr.	29.5 (28.4–30.5)	8.5 (7.9–9.1)	8.4 (7.8–9.0)	18.6 (17.9–19.8)
	30+ gr.	20.2 (18.6–21.9)	8.2 (7.9–8.5)	8.2 (7.9–8.5)	14.1 (13.0–15.3)
Average monthly price (95% CI)	Per cigarette	38.0 (37.6–38.5)	35.2 (34.4–36.1)	34.9 (34.1–35.8)	39.8 (39.4–40.2)
	Per gram	32.5 (32.1–33.0)	30.5 (29.7–31.2)	30.6 (29.9–31.3))	33.6 (33.1–34.1)

**Table 2:** Adjusted mean differences between data sources not yet used in tobacco research and Nielsen Scantrack for price per cigarette and price per gram (May 2013–February 2017).

	a. Mean Difference in Price per Cigarette		b. Mean Difference in Price per Gram	
	Model 1	Model 2	Model 1	Model 2
Kantar retail	−0.005	−0.008	0.127	−0.247
(*p*-value)	(<0.001)	(<0.001)	(0.338)	(<0.001)
[95% CI]	−0.006 to −0.004	−0.008 to −0.007	−0.132 to 0.386	−0.384 to −0.110
Kantar online	−0.007	−0.004	0.180	−0.218
(*p*-value)	(<0.001)	(<0.001)	(0.175)	(0.002)
[95% CI]	−0.009 to −0.005	−0.005 to −0.003	−0.080 to 0.441	−0.356 to −0.080
Nottingham OPD	0.013	0.000	0.563	0.096
(*p*-value)	(<0.001)	0.210	(<0.001)	(0.057)
[95% CI]	0.012 to 0.015	−0.000 to 0.001	0.376 to 0.750	−0.003 to 0.195
Pack size	Yes	No	Yes	No
Time (year and month)	Yes	Yes	Yes	Yes
Product fixed effect	No	Yes	No	Yes
Observations	47,976	47,976	8552	8552
